# A Non-destructive Method to Quantify Leaf Starch Content in Red Clover

**DOI:** 10.3389/fpls.2020.569948

**Published:** 2020-10-15

**Authors:** Lea Antonia Frey, Philipp Baumann, Helge Aasen, Bruno Studer, Roland Kölliker

**Affiliations:** ^1^Molecular Plant Breeding, Institute of Agricultural Sciences, ETH Zurich, Zurich, Switzerland; ^2^Sustainable Agroecosystems, Institute of Agricultural Sciences, ETH Zurich, Zurich, Switzerland; ^3^Crop Science, Institute of Agricultural Sciences, ETH Zurich, Zurich, Switzerland

**Keywords:** red clover, starch content, hyperspectral imaging, partial least square regression, forage quality, grassland

## Abstract

Grassland-based ruminant livestock production provides a sustainable alternative to intensive production systems relying on concentrated feeds. However, grassland-based roughage often lacks the energy content required to meet the productivity potential of modern livestock breeds. Forage legumes, such as red clover, with increased starch content could partly replace maize and cereal supplements. However, breeding for increased starch content requires efficient phenotyping methods. This study is unique in evaluating a non-destructive hyperspectral imaging approach to estimate leaf starch content in red clover for enabling efficient development of high starch red clover genotypes. We assessed prediction performance of partial least square regression models (PLSR) using cross-validation, and validated model performance with an independent test set under controlled conditions. Starch content of the training set ranged from 0.1 to 120.3 mg g^–1^ DW. The best cross-validated PLSR model explained 56% of the measured variation and yielded a root mean square error (RMSE) of 17 mg g^–1^ DW. Model performance decreased when applying the trained model on the independent test set (RMSE = 29 mg g^–1^ DW, *R*^2^ = 0.36). Different variable selection methods did not increase model performance. Once validated in the field, the non-destructive spectral method presented here has the potential to detect large differences in leaf starch content of red clover genotypes. Breeding material could be sampled and selected according to their starch content without destroying the plant.

## Introduction

Temporary and permanent grassland account for roughly 70% of agricultural land and play a significant role in sustainable agriculture worldwide by providing roughage for ruminant livestock production. Pasture and grassland-based agroecosystems maintain carbon balances, nutrient cycles, biodiversity and water quality (Steinfeld et al., 2006). However, they were gradually replaced by intensified production systems, where the high feed energy content required by today’s livestock breeds is largely covered through starch from cereals and maize. Starch is an important form of assimilated carbohydrates in plants, which diurnally accumulates in the leaf and is nocturnally mobilized to support growth (Geiger and Servaites, 1994; Stitt et al., 2007; Stettler et al., 2009). The accumulation of starch and its linear degradation at night is thought to be crucial for stable growth and to be directly correlated to plant biomass (Graf et al., 2010; Mugford et al., 2014). However, plant biomass and leaf starch content are not always negatively correlated in species such as birdsfoot trefoil (*Lotus japonicus* L.) or red clover (*Trifolium pratense* L.; Vriet et al., 2010; Ruckle et al., 2017). Starch accumulation and degradation varies not only among plant species, but also among genotypes, seasons, and management regimes (Griggs et al., 2007; Pelletier et al., 2010; Moraes et al., 2013; Liu et al., 2018).

Red clover is one of the most important forage legumes in temperate climates (Taylor, 2008). Its high yield potential, high crude protein content and high digestibility make it an excellent feed, not only for cattle but also for other livestock and poultry (Broderick, 1995; Halling et al., 2001). Red clover has the potential to accumulate up to one third of its leaf dry mass as starch, and some genotypes degrade less than 50% of their starch during the night (Ruckle et al., 2017). Thus, selecting for red clover plants with high starch content and low degradation rates is likely to result in high starch cultivars. These could provide an alternative, high-energy feed source, which would significantly improve sustainability of ruminant livestock production.

Developing a high starch red clover requires a better understanding of the starch metabolism in red clover and an efficient method to quantify starch in leaf tissue. Starch is commonly quantified with an enzymatic method, where leaf samples are flash frozen, ground and weighted before extraction is performed (Hostettler et al., 2011). This procedure is laborious, expensive and involves destructive sampling. A non-destructive method to measure leaf starch content would enable detailed studies of starch turnover in red clover plants, and dynamic changes during plant development could be traced on the same plant throughout the entire season. Specifically, different genotypes could be investigated under different management regimes and across different environments over an extended period of time. Furthermore, this method could be applied in breeding to develop high starch red clover cultivars.

Hyperspectral imaging and near infrared spectroscopy (NIRS) are routinely used to estimate biochemical compounds such as lignin, cellulose, starch, sugars and proteins in numerous crops (Goetz et al., 1990; Hattey et al., 1994; Yoder and Pettigrew-Crosby, 1995). These two methods have largely replaced wet chemistry as the standard analytical procedure for detection and quantification of plant biochemical compounds in the food industry (Card et al., 1988; Barton, 1991). Infrared spectra result from the fundamental vibrational absorptions of photons in the mid-infrared region (500–4000 cm^–1^, 350–25000 nm) by bonds within specific functional groups of molecules. These absorptions are mirrored to the NIR region (Card et al., 1988). Multivariate statistics, chemometrics, or machine learning methods are then used to quantify and classify specific compounds or properties (Kumar et al., 2001). NIRS or other spectral techniques are most accurate when using dried and homogenized (i.e., milled) plant material. For example, starch has been accurately quantified on dried cotton leaves or dry forage maize using NIRS (*R*^2^ > 0.9; Hattey et al., 1994; Hetta et al., 2017; Lu et al., 2017). Estimating chemical compounds with spectral measurements on fresh leaf tissue is often less reliable due to masking effects of light absorption by the cuticle or the leaf water content (Curran et al., 1992; Fourty and Baret, 1998). For successful spectroscopy-based diagnostics using fresh leaf tissue, spectral pre-processing and statistical modeling are essential to at least partially correct for confounding effects (Curran et al., 1992).

The following study aimed at developing a non-destructive spectroscopic method to estimate leaf starch content in fresh leaf tissue of red clover. Although developed in the greenhouse under controlled conditions, such a method could, once validated in the field, enable to monitor starch turnover on the same genotype over a longer period, under different management regimes, and under various environmental conditions.

## Materials and Methods

### Plant Material and Growth Conditions

Leaf starch was determined in two independent sets (i.e., a training set and a test set) of plants grown in two separate experiments. Plants from the two experiments were grown in a completely randomized design. The plants of the test and the training set were grown in spring and autumn 2018, respectively. All plants were clonally propagated using cuts that contained only one shoot and one root meristem to ensure comparable physiological states of all plants. These clonally propagated plants were grown in a climate chamber for 90 days before harvesting (pot size ø 16 cm, height 13 cm; 3:2:1 soil: peat: perlite substrate; photoperiod of 14:10-hour L:D; day temperature 20 ± 2°C, night temperature 15 ± 2°C; relative humidity, 60 ± 10%; fluorescent light bulbs T7 (Phillips, DE), and Grolux^®^ fluorescent lamps (Sylvania, DE) at a ratio of 3:1; total light intensity 200 μmol m^–2^ s^–1^). Samples were taken at the end of the night (EN; before lights were turned on), and at the end of the day (ED; before lights were turned off). Single plants were selected from cultivars from Europe and from breeding germplasm. In addition, nine plants from an advanced breeding population previously shown to have a high variation for leaf starch content, were selected (Ruckle et al., 2017; Supplementary Table S1). Wet laboratory measurements were taken on exactly the same material as used for the spectral measurements.

**FIGURE 1:**
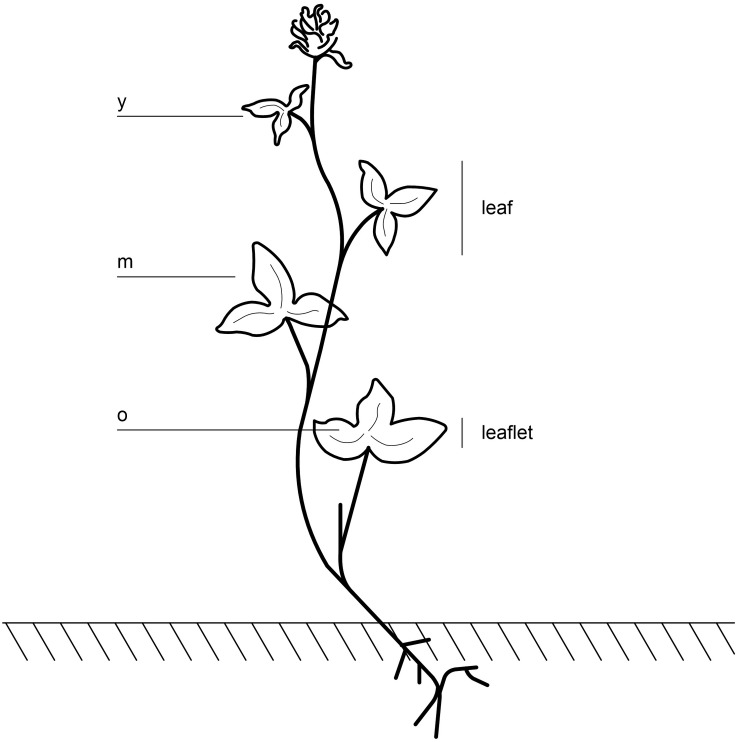
Sampling on red clover plants was performed on the youngest leaf, where three leaflets were fully emerged (y), on the three leaflets of the oldest leaf (o), and on three different mature leaves, in total nine leaflets (m).

The training set included 18 genotypes, six thereof clonally duplicated (Supplementary Table S1). Starch was measured on 15 leaf cuts per plant, taken on the three leaflets of each leaf. The youngest, fully emerged leaf (y), the oldest leaf (o) and three mature leaves (m) were sampled (Figure 1), resulting in 360 measurements. The test set included six genotypes; three genotypes were harvested at ED and the same three genotypes at EN (Supplementary Table S1). Ten leaf cuts were taken per plant on mature leaves, resulting in 60 measurements.

### Leaf Spectroscopy

Leaflets were cut using a round, sharpened tube with a diameter of 12 mm to standardize leaf area (Supplementary Figure S1). These leaf cuts were placed on the matt black surface of the FieldSpec 4 pro device (Analytical Spectral Devices, Boulder, CO, United States; Ely et al., 2019). The device is not influenced by external light sources, potentially enabling the application in field experiments. Radiance between 350 and 2500 nm was measured. The spectrometer’s contact probe was fixed on a clamp, and the sample was placed so that no light escaped through the sides. Leaf samples were referenced to a spectralon white reference every fifth recording and the radiance measurements where transformed to reflectance. Immediately after taking spectral measurements, leaf cuts were flash frozen in liquid nitrogen and freeze-dried for 48 h.

After taking spectral measurements, whole plants from the training set were cut 2 cm above ground, flash-frozen and freeze-dried for starch quantification.

### Wet Lab Analysis for Starch Quantification

Starch in leaf cuts and whole plants was quantified using a protocol of Hostettler et al. (2011), which was modified and described by Ruckle et al. (2017). Two additional clones of one genotype, not included in the correlation model, were iodine stained to visualize the starch pattern within a plant. Plants were harvested either at ED or at EN, washed with tap water and placed in 80% (v/v) boiling ethanol. After 2 h, when plants were transparent, they were removed and placed in Lugol’s solution. After 10 min, the Lugol’s solution was rinsed off to destain the non-target areas. The plants were photographed on a light-table (Hostettler et al., 2011).

### Statistical Analyses

Statistical analyses were performed using the R statistical software version 3.6.0 (R Core Team, 2019). As assumptions of normality of residuals were not met, an exact Wilcoxon rank sum test was chosen to detect differences between harvest times, and a Kruskal-Wallis test for multiple pairwise comparison at α = 5%.

### Pre-processing of Spectral Data

Spectral analysis was realized using the R package simplerspec (Baumann, 2019). The mean reflectance values of 10 measurements per sample were used. Leaf spectra were pre-processed prior to modeling. Gaps between the different detector arrays at λ = 1000 and at λ = 1800 nm were splice corrected. Spectra were smoothed with the Savitzky-Golay first derivative filter using a 3rd-order polynomial at a 21-point window (21 nm at a resampled spectrum interval of 1 nm; R package prospectr (Stevens and Ramirez-Lopez, 2020). Spectral pre-processing is crucial to reduce significant noise and baseline drift resulting from light scatter before establishing a correlation model. After smoothing the spectra with Savitzky-Golay, the spectral variables were centered and scaled prior to relating them to leaf starch using partial least squares regression (PLSR), in order to consider variables equally independent of their variation in absolute values. PLS regression is a substantial chemometric method, which can cope with multicollinearity in spectra and delivers robust calibration models with many predictors and few observations (Zhao et al., 2015). To further reduce collinearity in processed spectra, only every forth wavelength was kept for modeling, resulting in 533 spectral predictor variables.

### Model Development

Leaf reflectance data from the training set was modeled by PLSR with the orthogonal scores algorithm (also NIPALS; Wold et al., 1983), using the pls R package (Mevik et al., 2020). Separate models were developed with raw or pre-processed spectra as predictors. A 5-times repeated 10-fold cross-validation scheme was used to fit the models, to determine the best number of components (ncomp), and to estimate model performance of the final model. A constant random seed was set for resampling, yielding identical hold out data across all models. Model reliability was assessed by the coefficient of determination (*R*^2^) and the slope (*b*) of a linear regression with intercept, by the root mean square error (RMSE, Eq. 1), by the bias or mean error (Eq. 2), and by the ratio of performance to deviation (RPD, Eq. 3). The evaluation metrics were calculated by aggregating all holdout predictions from the repeated 10-fold cross-validation (*y*_*i*_) and corresponding observed values (*y*_*i*_) grouped by ncomp.

(1) $$RMSE=\sqrt{\frac{\sum_{i=1}^{n}{\left(y_{i}-{\hat{y}}_{i}\right)}^{2}}{n}}$$

(2) $$Bias=\frac{\sum_{i=1}^{n}\left(y_{i}-{\hat{y}}_{i}\right)}{n}$$

(3) $$RPD=\frac{\text{sd}\left(y_{i}\right)}{\text{RMSE}}$$

Variable influence on projection scores (VIP) is a measure of variable importance tailored to PLS regression (Wold et al., 1993; Chong and Jun, 2005). VIP scores were calculated from the PLS regression parameters taking multicollinearity into account, which is likely to occur because of the nature of spectroscopic data. VIP scores are considered as a robust measure to identify relevant predictors, here important wavelengths. A variable with VIP above 1 contributes more than the average variable to the model prediction. The VIP value *vj* was calculated for each wavelength variable *j* as

(4) $$v_{j}=\frac{\sqrt{p\sum_{a=1}^{A}[SS_{a}{\left(w_{aj}/∥w_{aj}∥\right)}^{2}}}{\sqrt{\sum_{a=1}^{A}\left(SS_{a}\right)}}$$

where *w*_*aj*_ are the PLS regression weights for the a^th^ component for each of the wavelength variables and *SS*_*a*_ is the sum of squares explained by the a^th^ component (Eq. 4). The sum of squares *SS*_*a*_ for the a^th^ component was calculated from the score *q*_*a*_ of the predicted variable *y* and the *t*_*a*_ scores of the spectral matrix *X* (Eq. 5):

(5) $$SS_{a}=q_{a}^{2}t_{a}^{T}t^{a}$$

Variable influence on projection scores scores were also used to filter important predictors with a threshold of VIP > 1 within the training set, and the identified predictors were used to re-calibrate the test set and assess performance. This separation in the VIP filtering by independent tests was needed to avoid overfitting and over-optimistic assessment that typically occur when identifying subsets of features on the modeling data.

In addition to the VIP based filtering, two other procedures were applied for wavelength selection. First, the 50 most relevant wavelengths to estimate starch according to Pearson’s correlation coefficient (*r*) were taken to re-perform PLS regression. Second, four wavelengths that were assigned to starch in previous literature were taken and normalized with the reflection at the wavelength that had the smallest standard deviation across the entire wavelength range, prior to performing a multiple linear regression (MLR; Kumar et al., 2001).

### Model Evaluation Using the Test Set

The best training model tuned by cross-validation and refitted on all training data, and the training models with three different wavelength selection (filtering) methods were tested on the independent test data set (60 samples). The predictive ability of these final models was again evaluated using *R*^2^ and RMSE on the test set. Besides these test set predictions, a PLSR model was re-calibrated using only the test data. This re-calibration allowed to determine whether the test set possibly contained different or differently weighted spectral features relevant for starch prediction, so that PLSR training relationships did not generalize to this independent test experiment.

## Results

### Wet Lab Analysis for Starch Quantification

**FIGURE 2:**
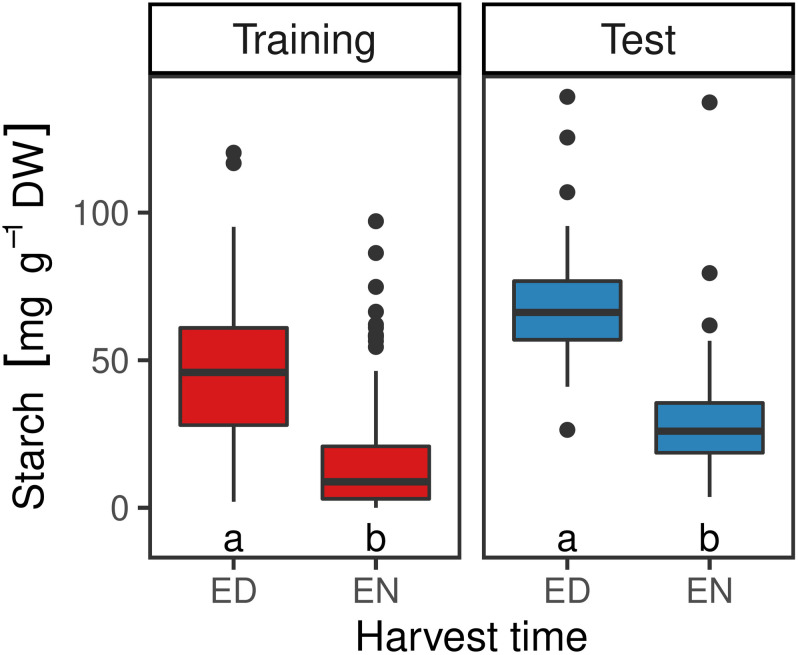
Starch content in mg g^–1^ DW in red clover plants of the training (*n* = 449) and the test set (*n* = 60) at the end of the day (ED) and at the end of the night (EN). Medians within the two sets with no letter in common are significantly different (Wilcoxon signed-rank test; α = 5%).

Starch concentration of samples of the training set harvested at ED ranged from 2.0 to 120.3 mg starch per g dry weight (DW), with a median of 46.3 mg g^–1^ DW. For the samples harvested at the end of the night (EN), starch concentrations ranged from 0.1 to 47.8 mg g^–1^ DW, with a median of 9.6 mg g^–1^ DW. Starch concentrations for the test set ranged from 26.41 to 125.44 mg g^–1^ DW for ED harvested samples, with a median of 66.18 mg g^–1^ DW. Plants harvested at EN had lower starch concentrations, ranging from 3.66 to 79.51 mg g^–1^ DW, with a median of 23.28 mg g^–1^ DW. Differences between samples harvested at ED and EN were statistically significant (*p* < 0.5) for both sets (Figure 2). In order to test the reproducibility of the enzymatic method, three technical replicates of the 24 plants of the training set were analyzed, resulting in a standard error (SE) of 0.096 mg g^–1^ DW (data not shown). Also, there was no substantial difference in dry matter content (dry weight/fresh weight) observed between samples from different plants or sampled at different time points, indicating that water content *per se* was not responsible for the differences in starch content observed by spectral analysis (data not shown).

**FIGURE 3:**
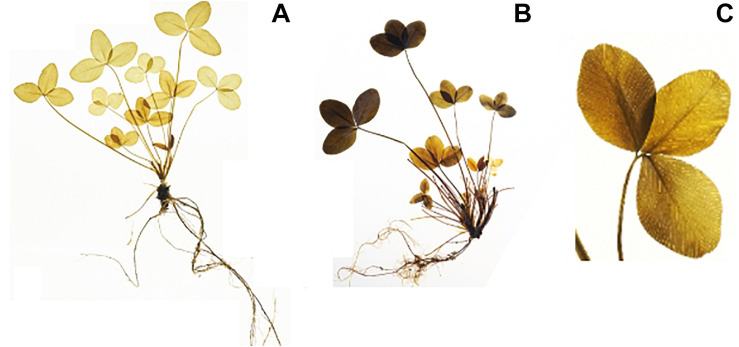
Iodine stained red clover plants (clones from the same genotype) visualizing starch distribution (dark coloration). There are clear differences in starch accumulation between harvest times **(A)** the end of the night (EN), **(B)** the end of the day (ED). Differences in starch accumulation are also visible within one plant **(B)**, and within one leaf **(C)**.

**FIGURE 4:**
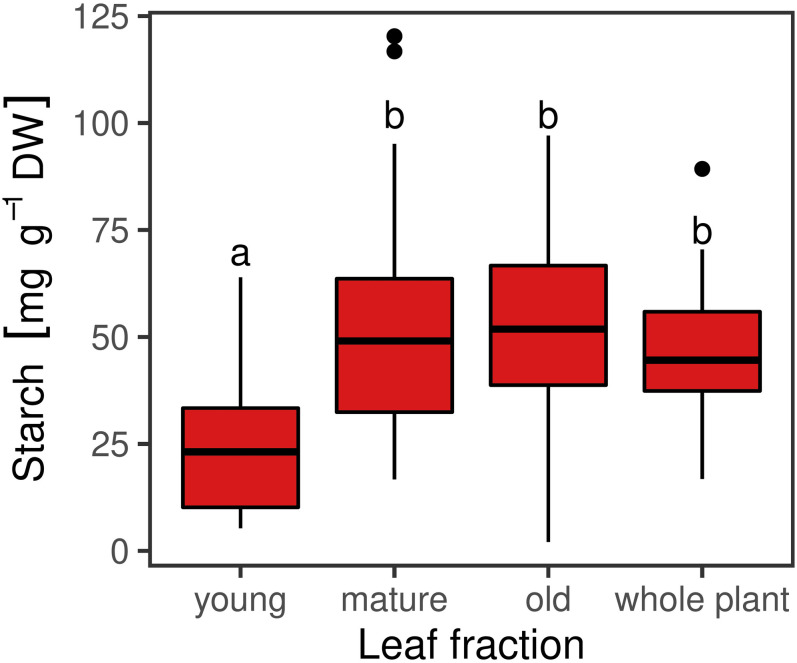
Starch concentrations in different leaf fractions [young (*n* = 35), mature (*n* = 108), old (*n* = 36)] and whole plants (*n* = 48) of red clover genotypes harvested at the end of the day (ED). Horizontal bars represent medians. Medians with no letter in common are significantly different (Kruskal-Wallis; α = 5%).

**FIGURE 5:**
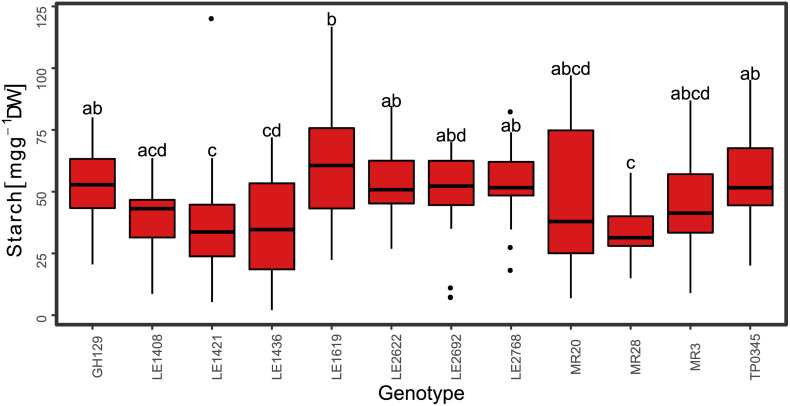
Starch content in mg g^–1^ DW for the red clover genotypes of the training set harvested at the end of the day (ED; *n* = 226). Medians with no letter in common are significantly different (Kruskal-Wallis, α = 5%).

The iodine stained leaves displayed analogous patterns. Plants harvested at ED showed higher starch accumulation than plants harvested at EN, indicated by a dark coloration of the leaves (Figures 3A,B). Differences in coloration were not only visible between diurnal time points, but also across and within leaves (Figures 3B,C). Dark coloration indicated that old leaves accumulated more starch than young ones. Coloration varied within a plant, showing a clear pattern with young leaves accumulating less starch compared to old leaves. While starch accumulation varied within one leaf, no clear pattern was distinguishable based on iodine visualization when observing starch accumulation within individual leaflets (Figure 3C). Observations in the iodine staining were confirmed by starch quantification in different leaf types. Young leaves had a significantly lower (*p* < 0.05) starch concentration when compared to mature leaves, old leaves or entire plants (Figure 4). Some differences between genotypes of the training set harvested at ED were statistically significant (*p* < 0.05), but a high variation within genotypes was observed (Figure 5).

### Spectral Measurements and Modeling

**FIGURE 6:**
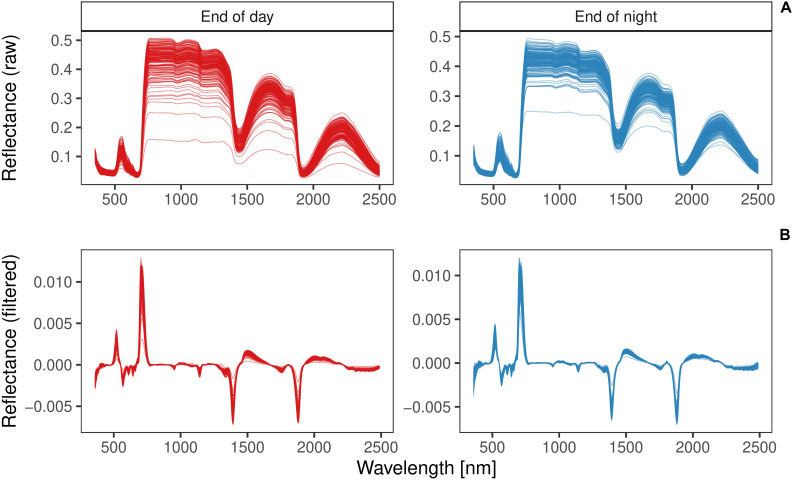
Raw **(A)** and Savitzky-Golay pre-processed reflectance spectra **(B)** of red clover samples of the training set (*n* = 338) harvested at the end of day (ED; red) or end of night (EN; blue).

The average reflectance spectra of the training set revealed similar patterns for both harvest time points ED and EN (Figure 6A). VNIR/SWIR (350–2500 nm) spectra had three main absorption regions, around the absorption bands of 700, 1400, and 1900 nm. Savitzky-Golay smoothed spectra showed very similar patterns across the entire wavelength range for samples harvested at ED and those harvested at EN (Figure 6B).

**FIGURE 7:**
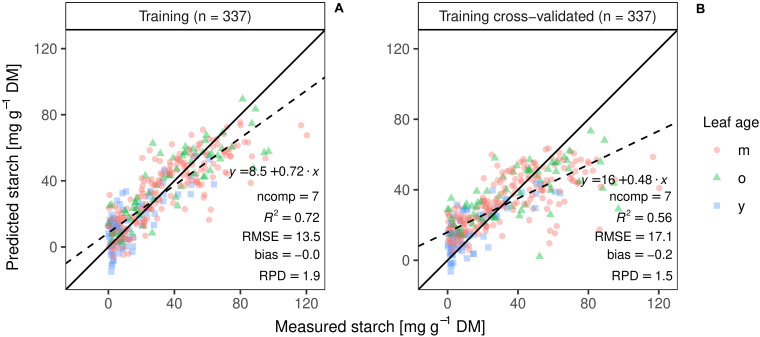
PLS regression of the training set **(A)** and best model performance of the cross-validation on the same set (**B**; ncomp = 9; *n* = 337) Different colors and shapes indicate the age of the leaves, m for matures leaves, o for the oldest leaf, and y for the youngest fully emerged leaf. Regression line (dashed line), 1:1 line (solid black line) and summary statistics are shown.

The best PLSR training model with pre-processed spectra resulted in an accurate starch prediction for the training set (*R*^2^ = 0.72, RMSE = 13 mg g^–1^ DW, bias = −0.0), using seven PLS components (Figure 7A). Five times repeated 10-fold cross validation performed on the same data set revealed a moderate correlation coefficient (*R*^2^_CV_) of 0.56, a RMSE _CV_ of 17 mg g^–1^, and a residual bias of −0.2 (Figure 7B).

Partial least square regression models modeling using pre-processed spectra performed better than modeling using raw spectra as predictors (Supplementary Figure S2). Separating cross-validated predictions by ED and EN resulted in lower correlation coefficients of *R*^2^_CV_ = 0.39 and *R*^2^_CV_ = 0.25 for ED and EN, respectively (Supplementary Figure S3). The starch estimates per individual plant had a wide range of *R*^2^_CV_ between 0 and 0.87 (Supplementary Figure S4). Including only the most relevant wavelengths for estimating starch content based on filtering training variable importance in the projection (VIP > 1) did not significantly improve prediction performance (Table 1). Prediction performance decreased compared to the full spectral model including only the best 50 with starch correlated wavelengths (PCC), and when using MLR with selected starch-assigned wavelengths (scaled), respectively (Table 1).

**TABLE 1:** Model performance using different filtering methods.

Model strategy	Training set		Test set		Description
	*R*^2^	RSME	*R*^2^	RSME	
VIP > 1	0.58	16.6	0.37	38.0	Filtering based on importance of the training variable in the projection (VIP)
PCC	0.53	18.5	0.37	32.0	Filtering based on the top 50 with starch correlated wavelengths
MLR	0.26	21.8	0.18	38.0	Reflectance at 556, 702, 1300, and 1960 nm divided by reflectance at 670 nm (minimum standard deviation)

*Model development using different filtering methods such as variable importance in the projection (VIP), the top 50 starch correlated wavelengths (PCC), multiple linear regression (MLR) before performing partial least square regression (PLSR). Best model performance of each filtering method determined by five time’s repeated 10-fold-cross validation was used to estimate leaf starch content of an independent test set.*

### Model Evaluation With an Independent Test Set

**FIGURE 8:**
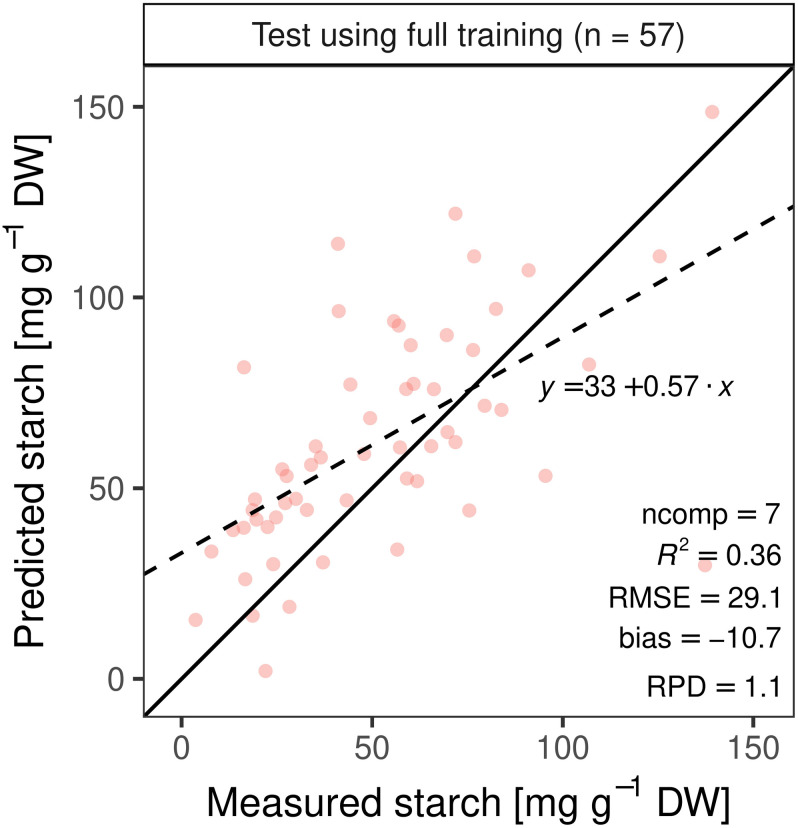
Best performing model from the training set was used to predict leaf starch content in an independent test set (*n* = 57).

Independent test set predictions (*n* = 57) using the best training PLSR model calibrated with pre-processed spectra (*n* = 337; ncomp = 7, all wavelengths) yielded a substantially lower *R*^2^ of 0.36 and larger RMSE of 29 mg g^–1^ DW (Figure 8). The three training models calibrated with variable selection (VIP > 1, top 50 correlations, and MLR with normalized assigned starch bands) resulted in inferior accuracy when applied to the test set (Table 1).

Model development using different filtering methods such as variable importance in the projection (VIP), the top 50 starch correlated wavelengths (PCC), multiple linear regression (MLR) before performing partial least square regression (PLSR). Best model performance of each filtering method determined by five time’s repeated 10-fold-cross validation was used to estimate leaf starch content of an independent test set.

## Discussion

Hyperspectral imaging on dry homogenized material is a widely used and well established technique, but applying this method on fresh plant material is not yet a standard analytical procedure (Card et al., 1988; Barton, 1991). Consequently, the correlation of hyperspectral measurements and wet lab results for starch reported in this study were clearly lower, when compared to NIRS measurements on dried plant material, where coefficients of determination (*R*^2^) reached 0.99 for nitrogen and starch contend of cotton leaves (Hattey et al., 1994). One challenge using fresh leaf material is the water in the fresh leaves. Liquid water is a strong absorber of the infrared radiation and predominant bands are in the regions near 1200, 1450, and 1950 nm (Fourty and Baret, 1998), where important wavelengths were present in this study (Supplementary Figure S5). It is therefore likely, that water absorption masked the absorption bands of starch molecules, impairing prediction of starch content to some extent (Kumar et al., 2001). Not only water absorption can obscure the starch absorption characteristics, but also the cell structure of fresh plants scattering light as it passes through multiple air and water boundaries. Furthermore, the distribution of starch in fresh leaves is not uniform with respect to the organization of cells and organelles (Kumar et al., 2001). The problems associated with the prediction of starch content in fresh leaves might be reduced, if spectral data is pre-processed (Wold et al., 1993). Indeed, pre-processing of the spectra considerably improved predictive accuracy compared to unprocessed reflectance spectra (Supplementary Figure S2), by removing systematic variation in spectra such as light scattering and thereby increasing the signal to noise ratio (Kuhn and Johnson, 2013).

Total starch concentration of the plant material in the training set was between 0.2 and 12% for plants harvested at ED and ranged from 0.01 up to 5% for the plant material harvested at EN. The starch concentrations of the training set were slightly lower than the concentrations of the test set. The total starch concentration was substantially lower than the ones published by Ruckle et al. (2017), where leaf starch concentration ranged from 6 up to 35% for ED harvested plants. This difference occurred most likely due to different growing conditions, since light and temperature have a high impact on starch accumulation.

Starch content in plant leaves typically varies in a diurnal pattern (Holt and Hilst, 1969). Starch is an important form of assimilated carbohydrates in forage legumes, which is accumulated in the leaf during the day and mobilized during the night to support growth (Stitt et al., 2007). Many studies have shown that starch contents highly depend not only on the diurnal cycle, but also on weather conditions, cutting time, plant fraction, and genotypic variation (Holt and Hilst, 1969; Geiger and Servaites, 1994; Graf et al., 2010; Claessens et al., 2016). In our study, an over 3-fold difference in starch content was observed between ED harvested plants and plants harvested at EN (Figure 2).

Mean genotypic differences for the training set ranged from 31.6 to 59.7 mg g^–1^ DW for the ED harvested plants (Figure 5) and from 2.2 to 37.6 mg g^–1^ DW for the EN harvested plants (data not shown), respectively, showing high variation within genotypes. The best PLSR training model explained 56% (*R*^2^ = 0.56) of the measured starch variation with an RMSE of 17 mg g^–1^ DW. The ratio of performance to deviation (RPD) followed the trend indicated by *R*^2^ values (Figures 7, 8 and Supplementary Figure S2, S3). The cross-validated overall bias was almost zero for the training set, while predictions on the test set had a bias of −10.7 mg g^–1^ DW.

These results imply that the developed vis–NIR PLSR model can predict differences between harvest time points and differences between extreme genotypes (Supplementary Figure S4). Nevertheless, 56% of starch variation explained by our model is lower than the proportions reported by Shorten et al. (2019). They used hyperspectral imaging systems (550–1700 nm) to estimate more than ten different quality compounds in perennial ryegrass (*Lolium perenne* L.). Low and high weight sugars were estimated separately and best model prediction for the high weight sugars using PLS regression resulted in an *R*^2^ of 0.68 and a RMSE of 19.9 mg g^–1^. Assigning two-third of the data to calibration and using the remaining data for validation resulted in a slightly lower model performance [*R*^2^ = 0.63 and RMSE of 21.6 mg g^–1^ (Shorten et al., 2019)]. Filtering spectral variables by a variable importance in the projection threshold (VIP > 1) did not considerably improve model performance (Table 1). This is in contrast to comparable studies where selecting important wavelengths improved model accuracy and reduced the redundancy effects of wavelengths, which had low weight in the model (Wold et al., 1993; Chong and Jun, 2005). Our results indicate that restricting PLSR with a subset of important spectral variables is not sufficient to estimate starch with equal effectiveness compared to the full-range vis—NIR data, confirming that many spectral features are important for starch prediction. For example, the wavelengths near 550, 770, 850, 1440, 1920 nm, from 1650 nm to 1850 and 2160 nm had a relatively high model contribution for estimating starch content in the training set (VIP analysis, Supplementary Figure S5). The red-edge region around 700 nm, where a local maximum of the first derivative is located and which is typically indicative for chlorophyll, had relatively low model importance. However, adjacent wavelengths to the red-edge were moderately important. The highest VIP in the training set was around 550 nm. This region was shown to be the second most important region in the vis—NIR for the spectral estimation of total carbon, nitrogen, leaf mass per unit area, protein and nitrate from wet leaves of 8 crop species (Ely et al., 2019). Starch absorbance in fresh leaves was further associated with wavelengths in the regions of 556, 702, 1300, and 1960 nm (Curran et al., 1992). These absorptions partly corresponded with the VIP patterns across wavelengths for the data of the present study. In addition, performing explanatory inference for spectra-model-compound linking is hampered by spectral overlaps due to dominant water bands and signals of other compounds related to starch. In fact, plant leaves contain many biochemical compounds with vis—NIR absorption regions that overlap with starch absorptions, or whose concentration directly or indirectly correlate to starch, such as cellulose, water or lignin, all having signals from O-H vibrations in the regions around 1450 and 1940 nm. Curran et al. (2001) performed both a correlative and stepwise regression analysis between 12 abundant structural, productive and storage compounds, and vis—NIR first derivative spectra of ground and dried slash pine needles. Among the components tested, starch exhibited the lowest coefficient of determination with first derivative spectra, and selected starch wavelengths were 1208, 1418, and 2172 nm, whereas 978 and 1208 nm were linked to starch absorption features. Native plant starch consists of a variable ratio of amylose and amylopectin. Amylose content in various mixtures was accurately discriminated with vis—NIR reflectance, showing major spectral feature differences between 1700 and 1800 nm in the pure form (Fertig et al., 2004). We found two VIP peaks with moderate importance (around 1.2), that might be linked to amylose and amylopectin signals. PLSR and the variable importance analysis were thus able to explain a significant proportion of the starch variability.

A model built from a single set of training observations is often not adequate to predict an independent data set (Naes and Martens, 1985; Kuhn and Johnson, 2013). If a model is tested on the same data that was used to fit the model, performance is often overestimated (Kuhn and Johnson, 2013). Our study showed that the cross-validated PLSR model underestimated high starch contents (Figure 7). The independent dataset from the second experiment (test set) allowed us to further validate model performance, in addition to cross-validation during training. As expected, the test prediction resulted in a 1.7-fold increase in RMSE (Figure 8). Moreover, models including only a subset of wavelengths were validated on the test set, resulting in a lower predictability (Table 1). The VIP analysis of the two independent sets (training and test) indicated that some important wavelengths regions occurred in both sets, but with different VIP magnitudes (Supplementary Figures S5, S6). For example, the absorption feature near 1450 nm was less important for the test set model fitting, compared to the model developed for the training set. Further, the training model had important features between 500 and 750 nm, whereas the re-calibrated test model had important wavelengths below 500 nm. These differences in VIP magnitudes and the additional regions relevant for prediction partly explain the poorer prediction performance of the test set when applying the training model. Despite the fact that two of the three genotypes from the test set were included in the training set, the spectra and models had only limited generalization capacity for starch contents.

Recalibration using only test data led to a slight decrease in RMSE compared to test prediction, but this substantially reduced bias. Thus, a new calibration may be needed for each independent trial or the current red clover starch spectral library needs to be augmented with more measurements from different independent trials with both genotypic and phenotypic variance in starch. Various environmental growth conditions influence starch accumulation and can thereby mask genotypic effects (Holt and Hilst, 1969). Based on the our results, we suggest follow up research that combines statistical methods to optimize knowledge transfer from such plant spectral libraries to new trials under substantial genotype x environment interaction. Thereby, the focus should be to find a trade-off between accuracy and the amount of new reference measurements needed, depending on the breeding application context. This library could be enlarged with data from new clover trials, so that it is continuously augmented with more genotypic and phenotypic variance in starch. We suggest to test methods from transfer learning research, which exploit different mechanisms to extract and transfer relevant information of collections of training data to new and partly related prediction tasks or application domains (Pan and Yang, 2010). For example, memory-based learning that constrains models based spectral similarity (Ramirez-Lopez et al., 2013), or data-driven search algorithms which filter relevant observations from spectral libraries that yield good performance on new local target samples (Lobsey et al., 2017) are candidate approaches that may be worth testing. We conducted all measurements under controlled conditions and leaves were completely removed from the plants. As a next step, it is crucial to evaluate the method under field condition.

Despite the relatively low prediction accuracy, performance of the best PLSR training model was sufficient to detect differences between red clover genotypes with very high or very low levels of starch content. Therefore, once validated in the field, the method may be valuable for in large-scale QTL studies in bi-parental populations based on strongly contrasting parental starch phenotypes. Furthermore, it has the potential to directly assist phenotypic selection in the breeding of high-energy red clover cultivars.

The success of breeding forage crops with increased energy content was previously demonstrated by breeding perennial ryegrass cultivars with high levels of water-soluble carbohydrates (WSC). These WSC cultivars can substantially increase animal performance and nitrogen use efficiency in pasture-based animal production systems (Rasmussen et al., 2009). Red clover and ryegrasses are often cultivated in mixtures, not only due to their attractive diet composition, but also due to the transfer of N between species. In addition, grass-clover mixtures require fewer pesticide and herbicide applications, and protect soils against erosion (Dhamala et al., 2016; McKenna et al., 2018). Therefore, high starch red clover cultivars in mixtures with high WSC ryegrasses appear a particularly promising option, which brings us one-step closer toward an environmental sustainable feed production meeting the high energy requirements of modern livestock production.

## Conclusion

This study is unique in developing and testing a non-destructive method to predict leaf starch content in red clover plants. The described method is suitable to differentiate between high and low starch content in red clover genotypes. Unfortunately, model performance is not sufficient to trace small changes in starch accumulation. Therefore, the method is only partially suited to monitor starch metabolism in detail or to investigate the effect of environmental influences or management regimes throughout an entire season on the same plant. We suggest follow up studies to enlarge the current red clover starch spectral library by means of additional measurements from different independent trials, covering both genotypic and phenotypic variation in starch and to validate the method under field conditions. Currently, the level of resolution is sufficient for the method to differentiate high variance in starch and thus, can be integrated into existing breeding programs to get a rough estimate on starch levels of different red clover cultivars under controlled conditions.

## Data Availability Statement

The datasets presented in this study can be found in online repositories. The names of the repository/repositories and accession number(s) can be found below: https://zenodo.org/record/3598699#.XhOLvdko_d4.

## Author Contributions

RK, BS, and LF conceived the study. LF and PB performed the research, analyzed the data, and drafted the manuscript. RK, BS, and HA assisted with data analysis, interpreting the results, and drafting the manuscript. All authors read and approved the final version of the manuscript.

## Conflict of Interest

The authors declare that the research was conducted in the absence of any commercial or financial relationships that could be construed as a potential conflict of interest.

## Abbreviations

DW: dry weight
ED: at the end of the day
EN: at the end of the night
MLR: multiple linear regression
PCC: top 50 starch correlated wavelengths
PLSR: partial least square regression
VIP: variable importance in the projection.
