# Supplementary figures and images for: A Non-destructive Method to Quantify Leaf Starch Content in Red Clover
Source: Front Plant Sci. 2020 Oct 15;11:569948. doi: 10.3389/fpls.2020.569948 (PMC7593268; doi:10.3389/fpls.2020.569948)

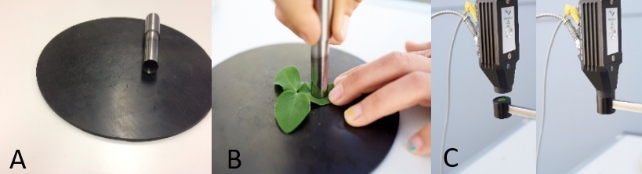

Supplement: Supplementary file 1 [file Image_1.JPEG]

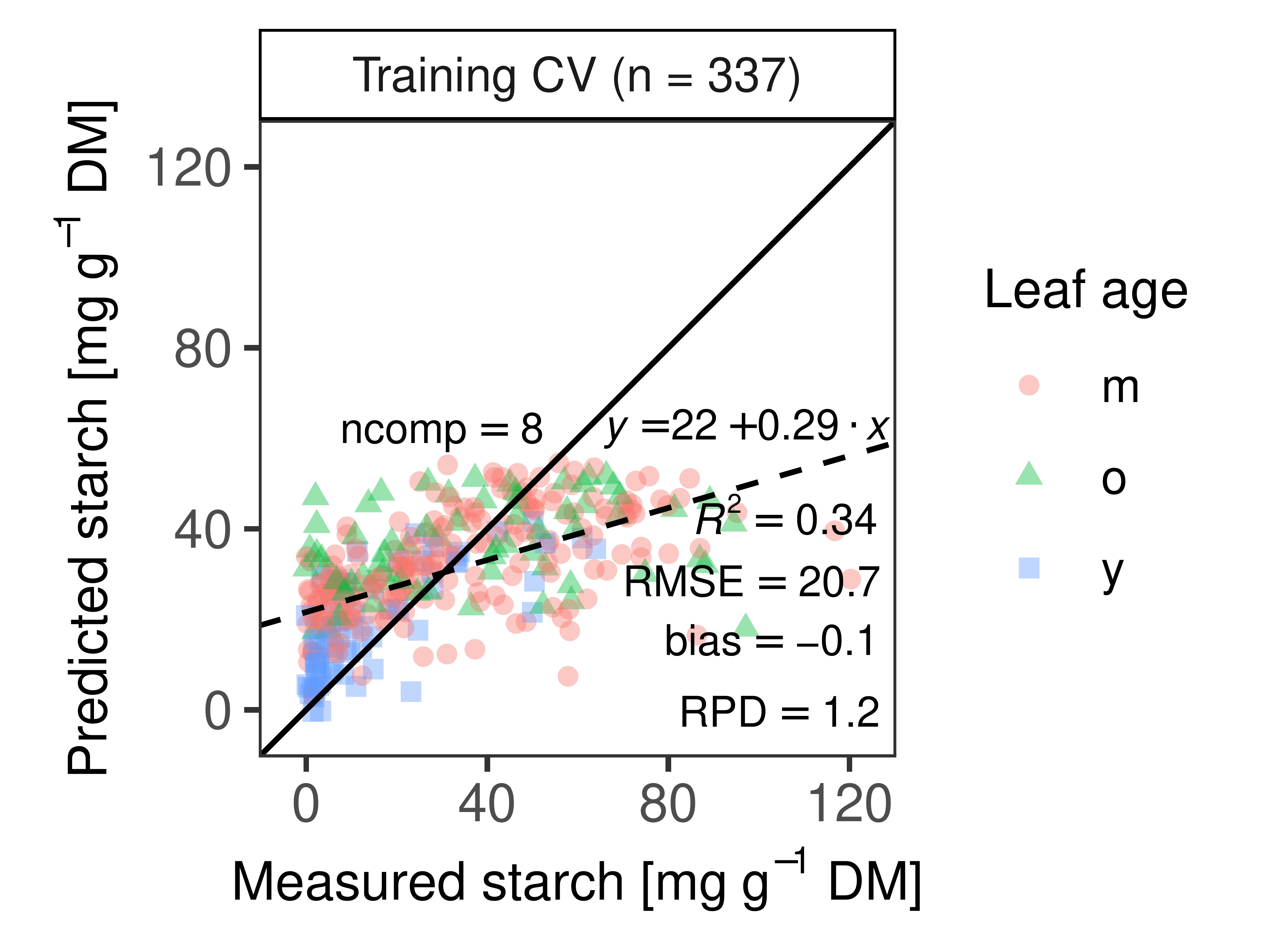

Supplement: Supplementary file 2 [file Image_2.JPEG]

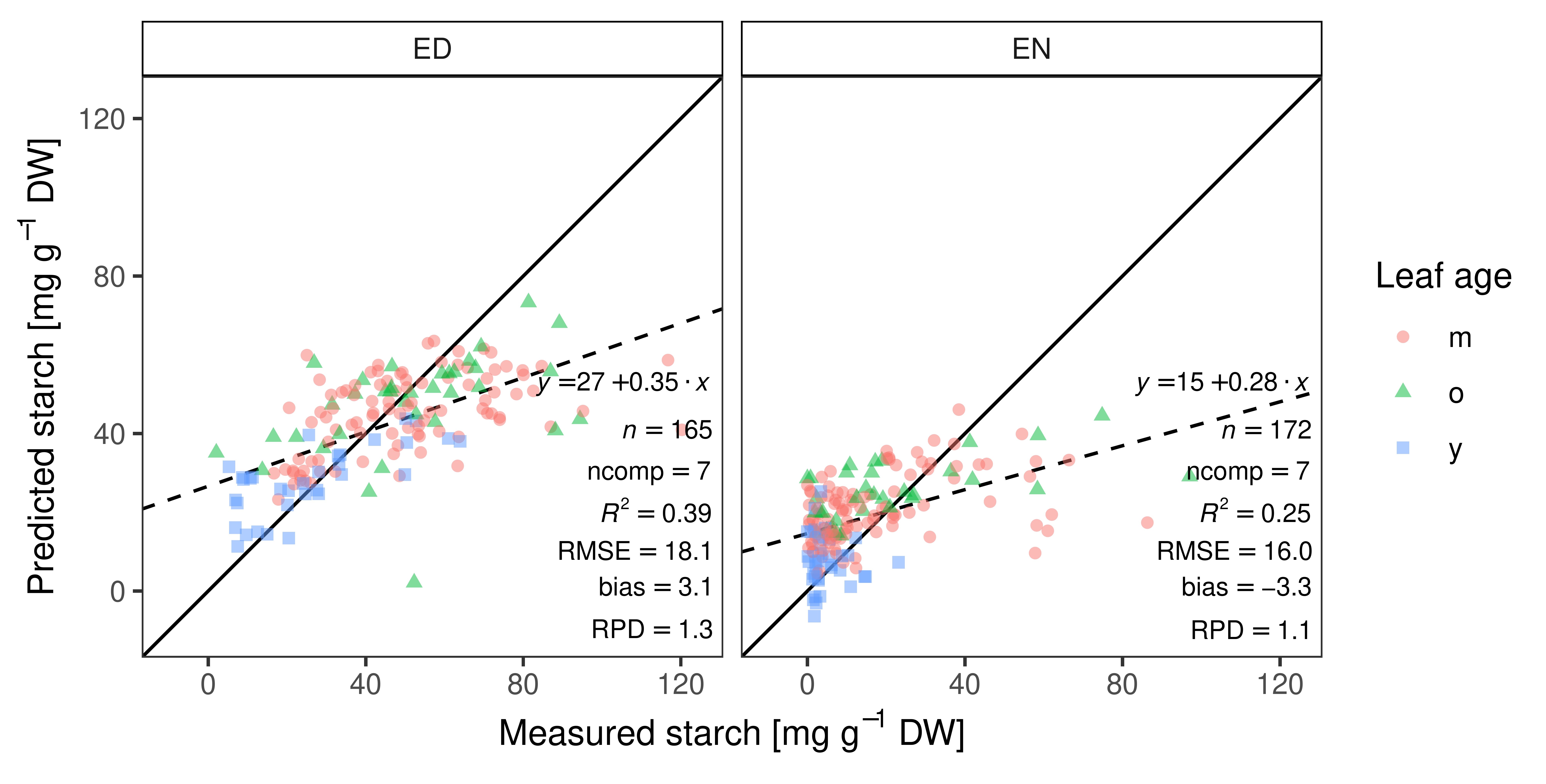

Supplement: Supplementary file 3 [file Image_3.JPEG]

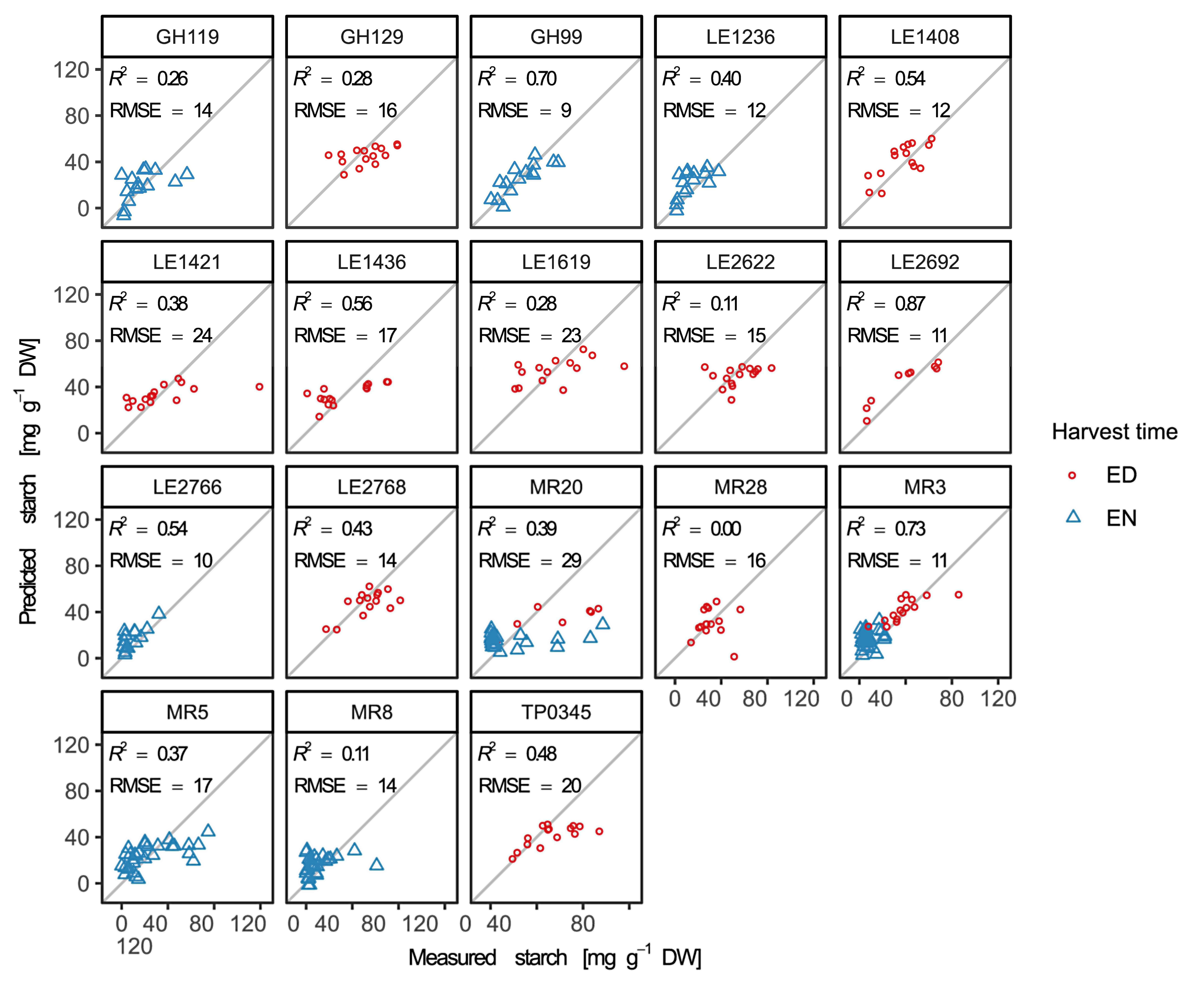

Supplement: Supplementary file 4 [file Image_4.JPEG]

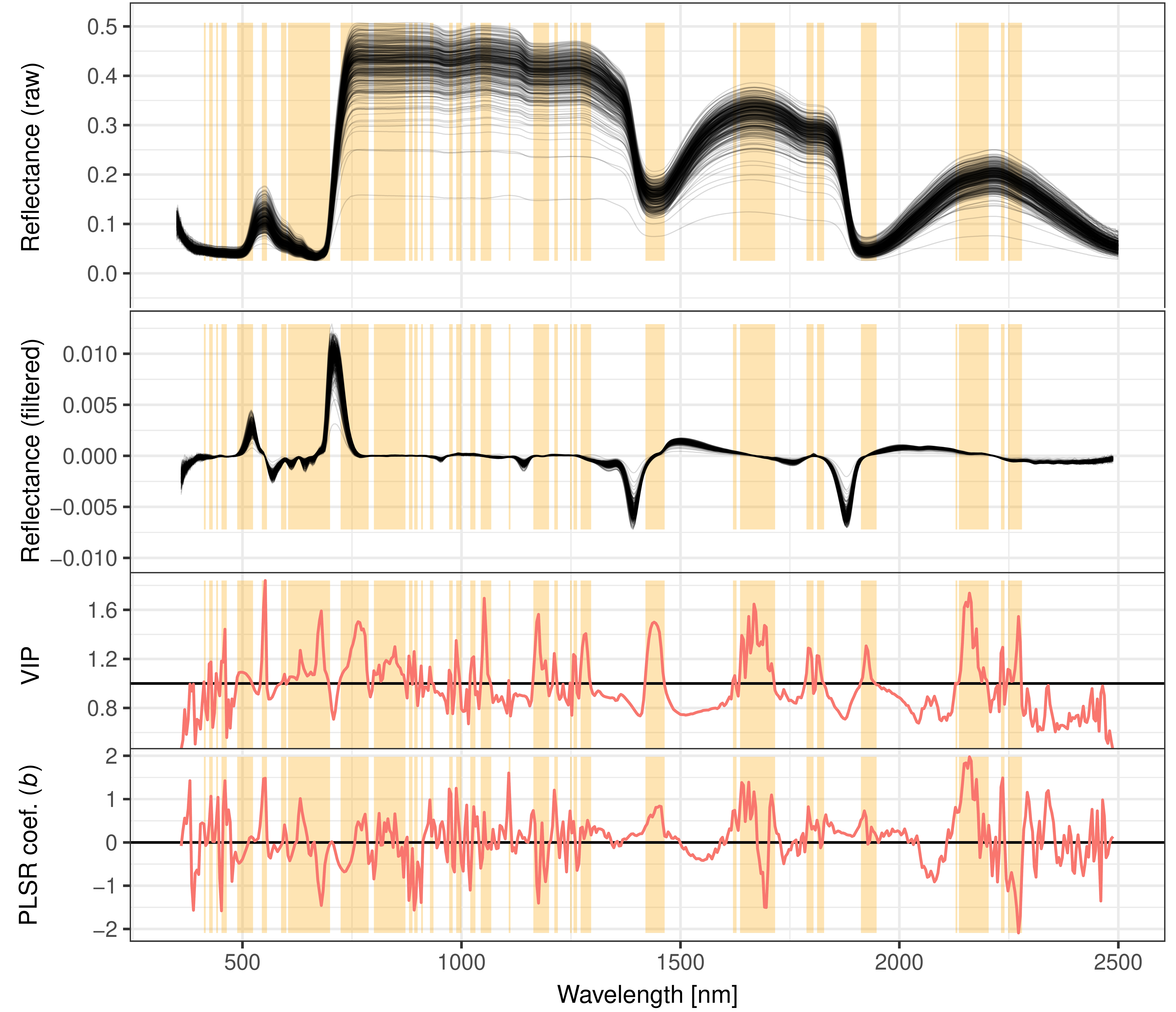

Supplement: Supplementary file 5 [file Image_5.JPEG]

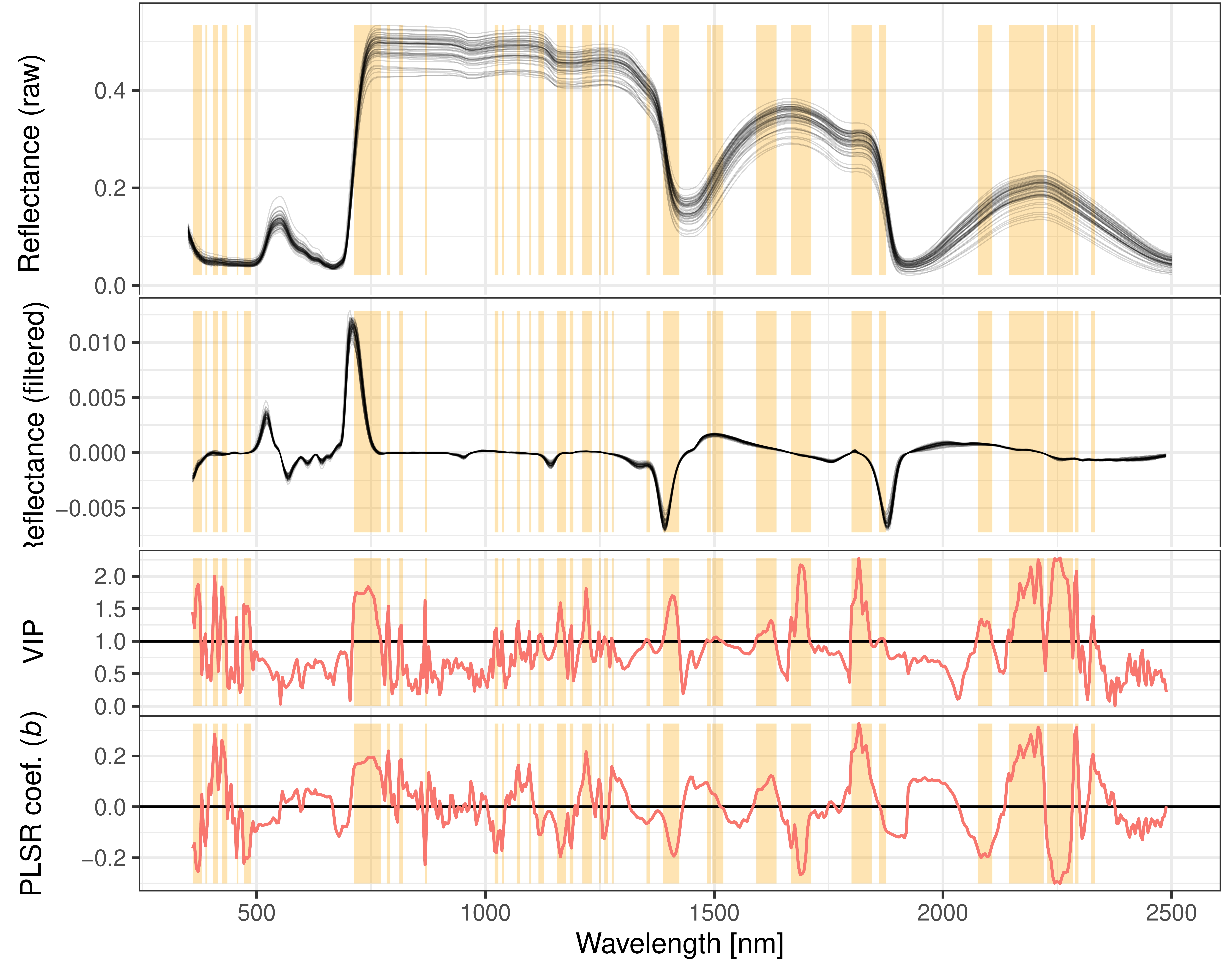

Supplement: Supplementary file 6 [file Image_6.JPEG]
