# Supplementary material for: A Non-destructive Method to Quantify Leaf Starch Content in Red Clover
Source: Front Plant Sci. 2020 Oct 15;11:569948. doi: 10.3389/fpls.2020.569948 (PMC7593268; doi:10.3389/fpls.2020.569948)
Supplement: Supplementary file 7 [file Table_1.docx]

Supplementary Material

# Supplementary Tables 1

Table S1 Name and origin of the red clover genotypes used in this study

| Plant from  cultivar / population | Gene bank accession number / germplasm ID, plant ID | Origin | Set | Time of measurement |
| --- | --- | --- | --- | --- |
| Albatros | LE1236 | France | Training | EN |
| Cremonese | LE2622 | Italy | Training | ED |
| Fox | LE1408 | France | Training | ED |
| Groninger Rode Klaver | LE1421 | Netherlands | Training | ED |
| Holitra | LE1436 | Germany | Training | ED |
| Ostpreußischer Rotklee | LE2766 | Germany | Training | EN |
| Piroschka | LE2692 | Hungary | Training | ED |
| Reichersberger | LE1619 | Austria | Training | ED |
| Remy | LE2768 | Germany | Training | ED |
| Breeding material | TP1245, GH99 | Switzerland | Training | EN |
| Breeding material | TP1245, GH119 | Switzerland | Training | EN |
| Breeding material | TP1245, GH129 | Switzerland | Training | ED |
| Breeding material | TP0345 | Switzerland | Training | ED |
| Breeding material | TP1245, MR3 | Switzerland | Training | ED / 2 x EN |
| Breeding material | TP1245, MR5 | Switzerland | Training /  Test | 2 x EN  ED / EN |
| Breeding material | TP1245,MR8 | Switzerland | Training /  Test | 2 x EN  ED / EN |
| Breeding material | TP1245, MR20 | Switzerland | Training | ED / 2 x EN |
| Breeding material | TP1245, MR28 | Switzerland | Training | ED |
| Breeding material | TP1245, MR31 | Switzerland | Test | ED / EN |

Name and origin of the *T. pratense* plant material used for the training and test set. Populations were obtained from IPK Gatersleben, Seeland, Germany (LE…) and Agroscope, Zurich, Switzerland (TP0345, TP1245). TP accessions from Agroscope are genetically diverse breeding germplasm, generated by open pollination of ten two twelve parental plants

## Supplementary Figure S1

Figure S1 (A) Round sharpened tube, used to cut out leaflets, (B) leaf cuts (C) and FieldSpec4 pro device opened and closed (D)

## Supplementary Figure S2

Figure S2 PLS regression of raw spectra and best model performance of the cross-validation (ncomp = 8; n=337). Different colors and shapes indicate the age of the leaves, m for matures leaves (red, circular), o for the oldest leaf (green, rectangle) and y for the youngest fully emerged leaf (blue, square). Regression line (dashed line), 1:1 line (fine black line) and summary statistics are shown.

## Supplementary Figure S3

Figure S3 PLS regression of the training set separated for the two different harvest times at the end of the day (ED; ncomp = 7, n = 165) and at the end of the night (EN; ncomp = 7, n = 172). Different colours and shapes indicate the age of the leaves, m for matures leaves (red, circular), o for the oldest leaf (green, rectangle) and y for the youngest fully emerged leaf (blue, square). Regression line (dashed line), 1:1 line (fine black line) and summary statistics are shown.

## Supplementary Figure S4

Figure S4 Prediction performance for each genotype of the training set

## Supplementary Figure S5

Figure S5 Reflectance spectra (top panel), pre-processed reflectance (Savitzky-Golay pre-processed; second panel), VIP filtering (third panel) and PLSR beta regression coefficients (bottom panel) for the training set

## Supplementary Figure S6

Figure S6 Reflectance spectra (top panel), pre-processed reflectance (Savitzky-Golay pre-processed; second panel), VIP filtering (third panel) and PLSR beta regression coefficients (bottom panel) for the test set

**
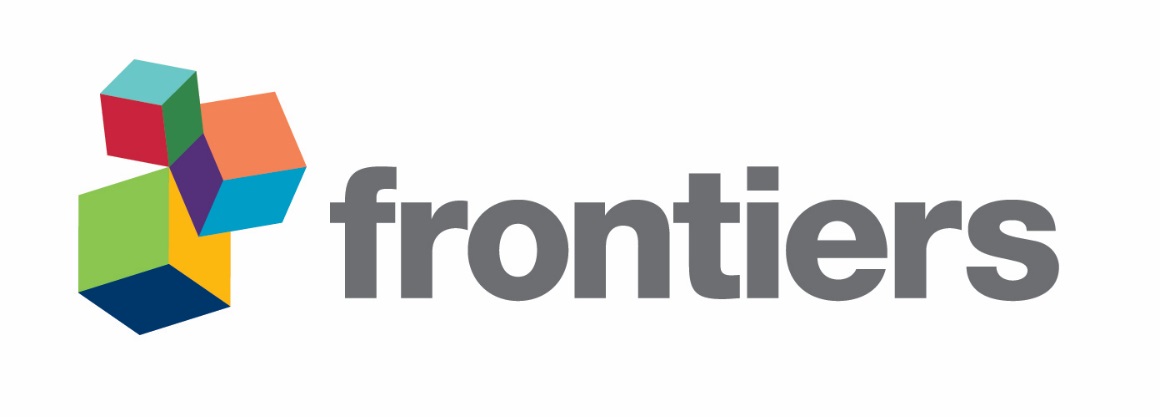
**
